# *Dermatophilus Congolensis* Infection of the Esophagus

**DOI:** 10.4021/gr216w

**Published:** 2010-07-20

**Authors:** Vivek S. Ramanathan, Alexander W. Jahng, Boris Shlopov, Binh V. Pham

**Affiliations:** a Department of Internal Medicine, St. Mary Medical Center, Long Beach, California, USA; b Department of Internal Medicine, Harbor-UCLA Medical Center, Torrance, California, USA; c Department of Pathology, Harbor-UCLA Medical Center, Torrance, California, USA; d Division of Gastroenterology, Harbor-UCLA Medical Center, Torrance, California, USA

**Keywords:** Dermatophilus, Congolensis, Esophagitis

## Abstract

We report the first case of *Dermatophilus congolensis* infection of the human esophagus. We demonstrate initial endoscopic diagnosis, progression and then spontaneous resolution of *D. congolensis* infection, once the patient's occupational exposure had ceased.

## Case Report

A 53-year-old female presented with two year history of chronic epigastric pain and ‘heart burn’. The review of systems was negative. The patient worked as a nail technician in close proximity to a horse race track, but she herself did not have direct contact with the animals. Physical examination and the labs were within normal limits.

Initial upper endoscopy showed slight granularity in the distal esophagus and superficial erosions in the antrum. Biopsy showed intestinal metaplasia and reflux esophagitis. *Helicobacter pylori* were negative. Six months later gastric mapping was performed to evaluate the intestinal metaplasia and showed acute-on-chronic erosive gastritis, but no dysplasia. Esophagoscopy revealed numerous 1-2 mm white plaques that was adherent from mid-esophagus down to the gastroesophageal junction (Fig. A). Brushings for cytology with Papanicolaou stain were done to rule out *Candida*, but instead showed septated and filamentous hyphae and coccoid forms of bacteria consistent with *Dermatophilus congolensis* (Fig. B). Candida was not cultured.

**Figure A:**
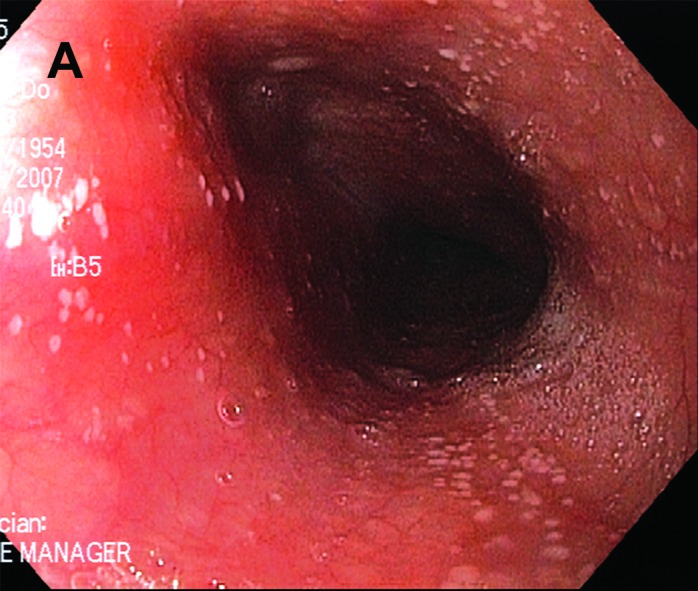
Esophagoscopy revealed numerous 1-2 mm white plaques that was adherent from mid-esophagus down to the gastroesophageal junction.

**Figure B:**
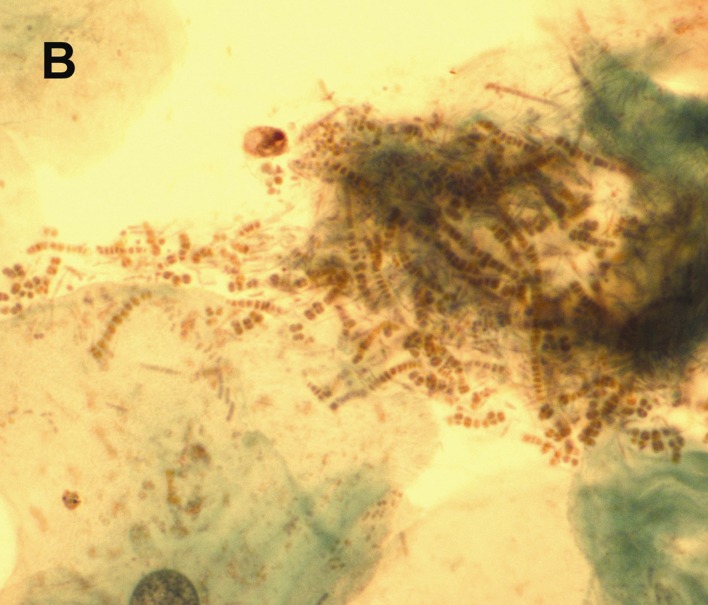
Brushings for cytology with Papanicolaou stain were done to rule out Candida, but instead showed septated and filamentous hyphae and coccoid forms of bacteria consistent with Dermatophilus congolensis.

Erroneously, the patient was then started on triple therapy for *H. pylori* by her primary care doctor which resulted in rapid and complete resolution of symptoms. Repeat endoscopy one month later showed progression of white plaques to involve the entire esophagus diffusely and was again consistent with *D. congolensis*. However, the patient continued to be asymptomatic but was unfortunately lost to follow-up before further recommendations could be made. Care was re-established one year later, six months after she quit her job. Final endoscopy showed complete resolution of white plaques despite lack of further intervention (Fig. C).

**Figure C:**
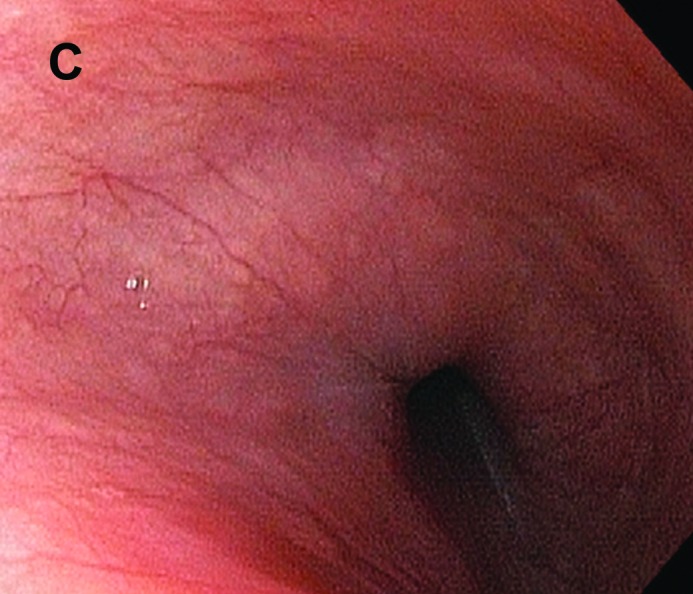
Final endoscopy showed complete resolution of white plaques despite lack of further intervention.

## Discussion

This is the first reported case of *D. congolensis* infection of the human esophagus. The only other reported case of human mucosal infection presented as leukoplakia [1]. *D. congolensis* is a gram positive, facultative anaerobe in the order *Actinomycetales* [2, 3]. It is a well known skin pathogen in animals that causes disease known as dermatophilosis; it causes proliferative and exudative epidermatitis. The most common mode of infection is thought to occur via direct transfer, possibly through areas minor trauma, or indirectly via ectoparasites. The human infection is rarer, and occurs in people with direct exposure to infected animals. It is generally self-limited with gradual and spontaneous resolution, though recurrent and chronic nodular forms have been noted. It is reported to be sensitive to variety of antibiotics *in vitro*, including to tetracycline, penicillin and macrolides. Treatment can be attempted but is often unsuccessful for two reasons; topical antibiotics fail to reach the deeper epidermis, which is further compromised by the exudative nature of the infection and systemic antibiotics fail because of the relative avascularity of the upper epidermal layer.

In this case report, we demonstrate endoscopic diagnosis, progression and spontaneous resolution of *D. congolensis* infection of the esophagus that was dissociated symptomatically. It is hypothesized that the infection occurred as part of occupational exposure, possibly through direct inhalation and swallowing of the contaminated air or fragmented nails of horse handlers at the race track, who frequented her place of work. The existing acid reflux with injured esophageal epithelium may have facilitated the inoculation and progression to infection. The progression of infection despite antibiotic therapy for *H. pylori* reflects the difficulty in delivering antibiotics to the site of infection. The spontaneous resolution after she quit her job may reflect the necessity for repeat exposure in maintaining the ongoing infection, or simply its natural cycle in humans.
